# Organothiol Monolayer Formation Directly on Muscovite Mica

**DOI:** 10.1002/anie.201913327

**Published:** 2019-12-18

**Authors:** Wester de Poel, Sander J. T. Brugman, Kim H. A. van de Ven, Anouk Gasseling, Jordi de Lange, Eleanor R. Townsend, Anthonius H. J. Engwerda, Maciej Jankowski, Melian A. R. Blijlevens, Ben L. Werkhoven, Jakub Drnec, Francesco Carlà, Roberto Felici, Aashish Tuladhar, Narendra M. Adhikari, James J. De Yoreo, Johannes A. A. W. Elemans, Willem J. P. van Enckevort, Alan E. Rowan, Elias Vlieg

**Affiliations:** ^1^ Radboud University Institute for Molecules and Materials Heyendaalseweg 135 6525AJ Nijmegen The Netherlands; ^2^ ESRF, 71 Avenue des Martyrs Grenoble France; ^3^ Utrecht University Institute for Theoretical Physics Princetonplein 5 3584 CC Utrecht The Netherlands; ^4^ Physical Sciences Division Pacific Northwest National Laboratory Richland WA 99352 USA

**Keywords:** calcite, monolayers, muscovite mica, organothiol, surface chemistry

## Abstract

Organothiol monolayers on metal substrates (Au, Ag, Cu) and their use in a wide variety of applications have been extensively studied. Here, the growth of layers of organothiols directly onto muscovite mica is demonstrated using a simple procedure. Atomic force microscopy, surface X‐ray diffraction, and vibrational sum‐frequency generation IR spectroscopy studies revealed that organothiols with various functional endgroups could be self‐assembled into (water) stable and adaptable ultra‐flat organothiol monolayers over homogenous areas as large as 1 cm^2^. The strength of the mica–organothiol interactions could be tuned by exchanging the potassium surface ions for copper ions. Several of these organothiol monolayers were subsequently used as a template for calcite growth.

## Graphical abstract

**Organothiols** with various functional endgroups could be self‐assembled into (water) stable and adaptable ultra‐flat organothiol monolayers on muscovite mica over homogenous areas as large as 1 cm^2^. The strength of the mica–organothiol interactions could be tuned by exchanging the potassium surface ions for copper ions.

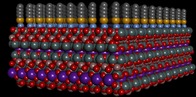

Self‐assembled monolayers (SAMs) of organothiols on solid gold substrates have become of major scientific importance since their discovery by Nuzzo and Allara in 1983.^1^ There are many other types of SAMs,^2^ but organothiols on gold are the dominant type with very well‐characterized properties and preparation methods.^3^ A wide range of applications exists for these layers, for example, as biosensors,^4^ to anchor proteins,^5^ to make nanometer thin sheets,^6^ to produce thin metal–organic frameworks,^7^ as substrates for crystal growth,^8^ and to produce nanopatterned arrays.^9^ For the majority of these applications a thin gold layer is used as a substrate for the growth of polycrystalline organothiol layers. To obtain such a surface, gold is usually evaporated onto muscovite mica, which is a mineral well‐known for its atomic flatness over large areas.^10^ However, gold surfaces that are atomically flat over these extended areas cannot be obtained, because atomic steps are unavoidable. Furthermore, the gold may still contain different domains, dislocations and impurities, which all contribute to a lower quality of the substrate, and thus also of the SAM. Moreover, gold may be etched by the organothiol,^11^ introducing even more inhomogeneities to the surface.

To circumvent these problems, we show that layers of a large variety of organothiols (Figure 1) can be grown directly onto muscovite mica (Figure 2) using a dip‐coating technique. In principle, these layers can be used for all the applications mentioned above, except when a conductive surface is required. A freshly cleaved muscovite mica surface is potassium‐terminated, but the ability to exchange the surface ions was exploited to make a copper‐terminated surface as well. Potassium‐terminated mica provides a surface with a hard metal (favoring ionic bonds), while copper terminated mica provides a soft metal (favoring covalent bonds) at the surface, providing a weak or strong interaction with the soft sulfur ligand (organothiol), respectively. The resulting layers were characterized with atomic force microscopy (AFM), surface X‐ray diffraction (SXRD) and vibrational sum‐frequency generation IR spectroscopy (SFG). Additionally, we investigated their application as templates for bio‐mineralization, specifically the growth of calcite, and compared the results to previously reported crystallization of this mineral on templating layers of organothiols on gold.

**Figure 1:**
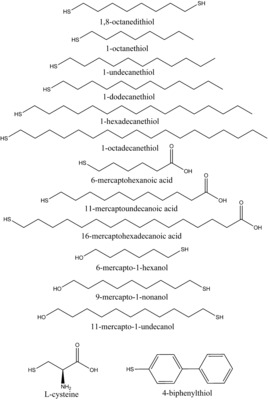
Chemical structures of the 14 investigated organothiol molecules. Those labeled with an asterisk form closed, flat layers.

**Figure 2:**
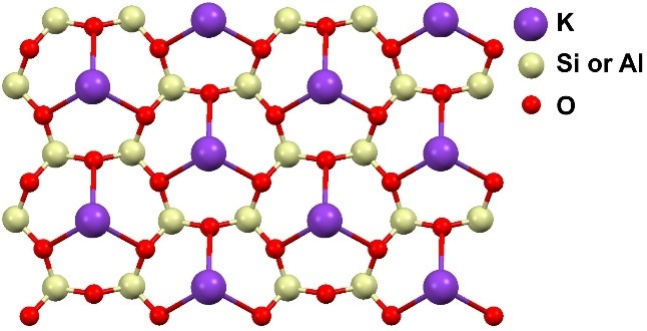
Top view of the structural representation of a muscovite mica (001) surface. 25 % of the silicon atoms are replaced by aluminum. A cleaved muscovite mica surface contains half of the amount of potassium ions shown to preserve charge neutrality.

AFM tapping mode images of closed layers of thiols on potassium‐terminated mica are shown in Figure 3 and the Supporting Information, SI‐1. The thickness of the layers was determined using imperfections, step edges, or by nanoshaving part of the surface and subsequently scanning a larger area in contact mode AFM (see Figure 3, Table 1; Supporting Information, SI‐1, SI‐2). Of the 14 investigated thiols, 9 formed closed and flat layers over the entire sample of 1 cm^2^, the other 5 formed incomplete layered structures when using the present preparation conditions (Figure 1).

**Figure 3:**
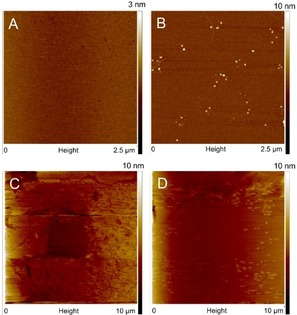
AFM height images of A) a typical flat monolayer of 1‐dodecanethiol on potassium‐terminated muscovite mica, B) a 16‐mercaptohexadecanoic acid layer on copper‐terminated muscovite mica, after being immersed in water for 90 hours. C) A nanoshaved area in a monolayer of 1‐hexadecanethiol on potassium‐terminated muscovite mica (first scan after shaving), and D) the same area after 126 minutes of continuous scanning, showing layer healing.

**Table 1:** Thickness of a selection of the organothiol layers on muscovite mica as obtained from AFM and SXRD measurements.^[a]^

Molecule	Mica termination	Thickness of topmost layer [nm] (AFM)	Layer thickness [nm] (SXRD)	% Calcite crystals	% (006)‐oriented calcite crystals	Epitaxy yes/no
None	K	–	–	67±29	15±4	no
11‐mercapto‐1‐undecanol*	K	0.6±0.2	0.7±0.1	93±2	30±7	yes
11‐mercapto‐1‐undecanol	Cu	1.7±0.5	1.0±0.1	94±3	38±11	yes
11‐mercaptoundecanoic acid*	K	0.5±0.2	0.7±0.1	99±1	15±3	no
11‐mercaptoundecanoic acid	Cu	1.8±0.6	0.7±0.1	99±1	12±1	no
1‐undecanethiol	K	0.6±0.2	1.6±0.1	61±1	27±2	no
1‐undecanethiol	Cu	0.5±0.2	1.0±0.1	74±19	37±10	no
l‐cysteine*	K	0.6±0.3	0.9±0.1	–	–	–
l‐cysteine*	Cu	0.5±0.3	0.3±0.1	–	–	–

[a] An asterisk indicates good agreement between both methods. Data on the crystallization of calcium carbonate on various substrates are provided (averaged over five samples from at least two crystallization batches).

Complementary to the AFM measurements, X‐ray specular diffraction data were used to determine the thickness of a selection of thiol layers (Table 1; Supporting Information, SI‐3). For all samples, the specular data were found to be different from that measured for the bare surfaces, confirming the presence of a SAM (Figure 4). For half of the layers (indicated by an asterisk in Table 1) the thickness measured using SXRD agrees well with that observed using AFM. A smaller AFM value compared to SXRD is likely caused by the fact that AFM detects only the topmost layer, while SXRD probes the full film. The cases where AFM gives the higher value are unexpected and may be due to local thickness variations: SXRD measures the average layer thickness over a large surface area (5 mm^2^), while AFM provides much more local information (scanning area ca. 100 μm^2^). Finally, the large measured thickness measured by AFM may also be an artefact of the flexibility of the SAMs compared to the mica substrate.

**Figure 4:**
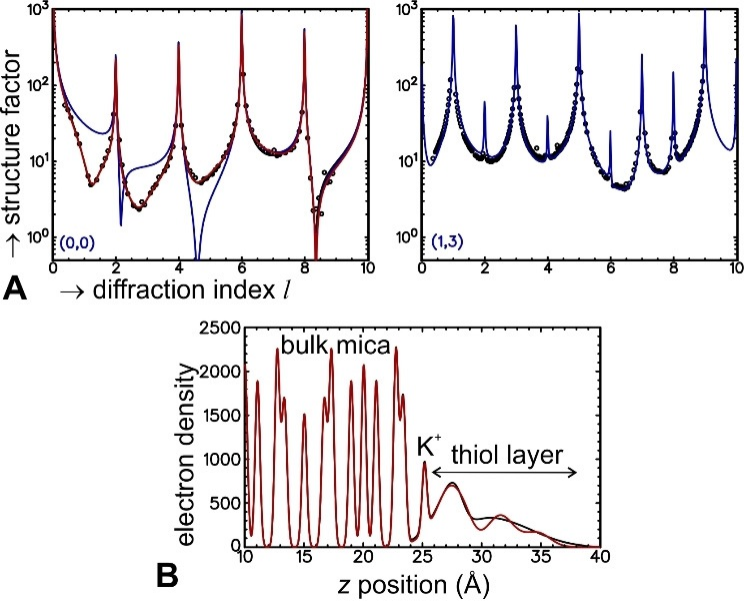
A) SXRD data (black dots) of a layer of 6‐mercaptohexanoic acid on K‐terminated muscovite mica. The labels in each graph indicate the *h* and *k*‐values for the specific crystal truncation rod. Blue line: fit based on bare K‐terminated muscovite mica, red line: fit based on K‐terminated muscovite mica with a (laterally disordered) layer of 6‐mercaptohexanoic acid. For the (13) rod, the red and blue lines are identical. B) Z‐projected electron density (electrons per unit cell) derived from the SXRD analysis. Black line: density from a generic model, red line: density derived from a model with 6‐mercaptohexanoic acid molecules.

The SXRD in‐plane data can also shed light on the lateral (dis)order of the organothiol layer in relation to the underlying mica surface. To this end, the specular, (1$\bar{1}$ ), (11), (1$\bar{3}$ ), (13), and (20) crystal truncation rods were measured. All the in‐plane rods for the surfaces covered with a thiol layer were essentially the same as for the bare surfaces (Figure 4; Supporting Information, SI‐3). This shows that the muscovite mica substrate remains intact, but more importantly also that the organothiol layers have no in‐plane order with respect to the mica substrate. It might be expected, similar to the gold–thiol interaction in the well‐ordered thiol‐on‐gold systems, that there is a metal–ligand interaction and associated ordering of the thiol sulfur atom with respect to the metal, but no such ordering was found by including this in the fitting model. The thiol layers are thus either crystalline and incommensurate or liquid‐like in the lateral direction. In‐plane scans along several directions did not reveal any evidence for in‐plane order of the organothiols either. Therefore, the investigated organothiol layers can in principle be compared to those created in a Langmuir trough, without any apparent influence of the crystalline substrate on the lateral organization of the molecules. However, in the present case the molecules are physisorbed to a solid support with which they interact, leading to a very flat layer (over macroscopic distances of 1 cm^2^) that can adapt itself to its environment. It was recently reported that thiols on gold also form a physisorbed bond,^12^ but in that case the bond is significantly stronger and the mobility (adaptability) of the thiols significantly less as demonstrated by the epitaxial relationship between gold and thiol.1b

Because of the disorder, the XRD data cannot reveal the local structure of the SAMs, but this can be achieved via IR spectroscopy.^13^ We therefore also performed vSFG IR spectroscopy measurements for 1‐undecanethiol and 11‐mercapto‐1‐undecanol (Figure 5). This not only confirms the presence of the layers, but also that the molecules are bonded to the surface through the thiol group, as proven by the presence of the CH_3_ stretching vibration (2880 cm^−1^) in 1‐undecanethiol (group points away from mica surface; Figure 5) and the absence of the S−H stretching vibration (ca. 2550 cm^−1^) for both 1‐undecanethiol and 11‐mercapto‐1‐undecanol (group is in contact with mica surface; Supporting Information, SI‐10). The fact that the ratio of CH_3_(ss):CH_2_(ss) is close to 1 indicates that the SAMs are disordered, thus corroborating the disorder derived from the XRD measurements. Data for Cu‐terminated mica indicates that the SAMs on this surface are more disordered (Supporting Information, SI‐10). Finally, wetting angle measurements (Supporting Information, SI‐11) confirm that the mica surface has been chemically modified, because with an applied SAM the angle is different from clean mica.^14^

**Figure 5:**
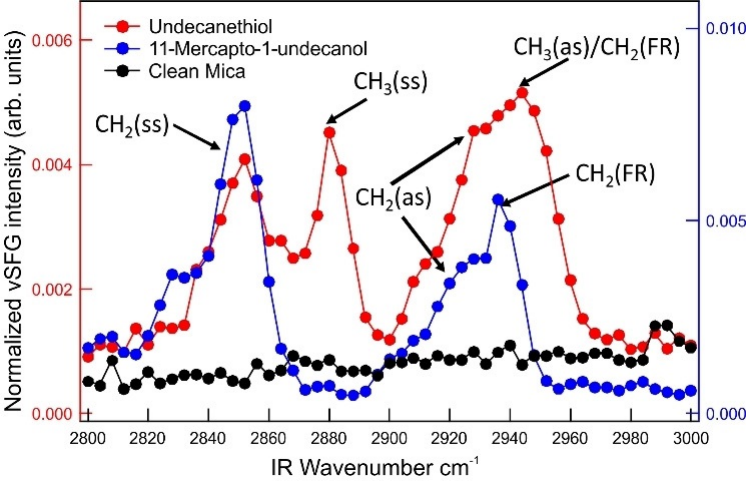
vSFG IR spectroscopy data with SSP polarization of K‐terminated mica with and without organothiol layers (see the Supporting Information, SI‐10 for details). (ss), (as), and (FR) stand for symmetric stretch, asymmetric stretch, and Fermi resonance, respectively.

The stability and mobility of the organothiol layers on mica were investigated with the help of AFM: aged samples were scanned to investigate stability, and partial layers and nanoshaved areas were continuously scanned to investigate layer mobility. The surface preparation procedure already reveals a hint about the stability of the thiol layers: they remained present after washing the muscovite mica crystal three times with 15 mL of dichloromethane. Furthermore, storage of the thiol‐covered surfaces at ambient conditions for 6 days left their surface morphology unchanged (Supporting Information, SI‐5). However, nanoshaved areas of thiol monolayers on potassium‐terminated mica showed healing (Figure 3). In the case of 1‐hexadecanethiol this healing occurred over a period of hours, while for shorter thiols no clearly demarked nanoshaved areas were visible, even though SXRD measurements showed that a monolayer remained present. This means that the shaved part was apparently rapidly refilled with molecules. Incomplete layers of 1,8‐octanedithiol and 9‐mercapto‐1‐nonanol on potassium‐terminated mica also showed rapid mobility, that is, the surface morphology changed in minutes (Supporting Information, SI‐6). This shows that these surfaces are adaptive compared to the rigid layers formed by thiols on gold surfaces.

We were able to tune the properties of the organothiol layers by exchanging the muscovite mica surface ions, thus altering the surface–organothiol interactions.^15^ Our choice of Cu^II^ is based on previously observed strong interactions between this ion and some organothiols (such as 6‐mercaptohexanoic acid) in aqueous solutions.^15^ Atomically smooth Cu^II^ ion‐exchanged layers can easily be obtained.^15^ With AFM flat layers were observed similar to those on potassium‐terminated muscovite mica (Supporting Information, SI‐7) and SXRD also showed no in‐plane order of the thiol layers (Supporting Information, SI‐3). In contrast to the thiol layers on potassium‐terminated mica, nanoshaved areas remained unchanged for at least 10–15 min for all the investigated organothiols (Supporting Information, SI‐8). This apparent immobility points to a stronger thiol–mica interaction, which we attribute to the presence of Cu^II^ ions on the surface. After submersion for 90 hours in water, AFM still revealed the presence of thiol layers on the surface, as was concluded from the height of imperfections (Figure 3; Supporting Information, SI‐5). The organothiol layers are thus remarkably water resistant, which is important for applications.

Previously, organothiol monolayers on gold have been used as templates for the controlled bio‐mineralization of crystals. Aizenberg et al.,^8^ for example, have used these templates to face‐selectively grow calcite crystals (CaCO_3_). To investigate the ability of our layers to serve as templates for crystallization, we grew calcite using organothiols of the same length (that is, 11‐mercaptoundecanoic acid, 1‐undecanethiol, and 11‐mercapto‐1‐undecanol) on both potassium‐terminated and copper‐terminated muscovite mica.

The crystallization results (Table 1) on the different surfaces were compared in terms of crystal size, number of crystals, polymorph selectivity (calcite, aragonite, or vaterite), orientation of calcite contact face relative to the surface normal of muscovite mica, and epitaxy (that is, whether calcite crystals have a specific azimuth with respect to the muscovite mica substrate).

11‐Mercaptoundecanoic acid and 11‐mercapto‐1‐undecanol layers promote the formation of calcite, (≥93 % abundance) and suppress aragonite and vaterite (Table 1). In the case of the other thiols, also various amounts of the other polymorphs of calcite were formed.

The nucleation density varies greatly for organothiols with different functional groups grown on gold,^8^ but this is not the case in our experiments (Supporting Information, SI‐9). The sizes of the grown calcite crystals, however, are in good agreement with those grown on organothiol layers on gold with the same functional group and exhibit the same trends for the matching functional groups.^8^

Aizenberg et al. found a clear relation between the crystallographic orientation of calcite crystals and the end‐group of the organothiol.^8^ For calcite crystals grown on our layers, no such relation was found (Figure 6 A). Apparently, most of the organothiol layers grown directly on muscovite mica are too disordered to achieve controlled oriented growth of calcite. Only monolayers of 11‐mercapto‐1‐undecanol grown on both potassium‐terminated and copper‐terminated mica reproducibly showed large areas of over 200×200 μm^2^ with a significant increase in the number of oriented and epitaxial calcite crystals (Figure 6 B). These crystals are oriented with the (006) plane parallel to the muscovite mica basal plane (Supporting Information, SI‐9), similar to the observations reported by Stephens et al. for calcite grown directly on mica.^16^ Although the lattice mismatch of calcite and muscovite mica is small (<4 %), we did not find epitaxially grown crystals on freshly cleaved mica. This shows that the 11‐mercapto‐1‐undecanol plays an important part in facilitating epitaxial growth. We speculate this may be the result of a specific layer density, the (limited) mobility of thiols within the layers, and the hydroxy endgroup of the thiol, or a combination thereof.

**Figure 6:**
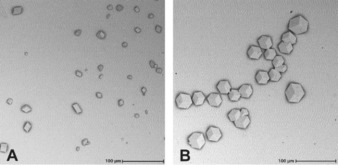
A) Scanning electron micrograph of randomly oriented calcite crystals grown on a layer of 11‐mercaptoundecanoic acid on Cu‐terminated muscovite mica, B) as in (A), showing an area with epitaxial calcite growth on a layer of 11‐mercapto‐1‐undecanol on Cu‐terminated mica.

The use of either potassium‐terminated or copper‐terminated muscovite mica as a substrate for the thiol layers had no significant effect on the calcite crystallization outcome, thus both layers are suitable.

In summary, a diverse array of organothiol monolayers can be grown directly onto potassium‐terminated and copper‐terminated muscovite mica substrates. The layers are (water) stable, flat over areas of 1 cm^2^, and their interaction strength with the substrate can be tuned by exchanging the muscovite mica surface ions. The interaction with the copper‐terminated surface is stronger, leading to less mobility and higher stability of the thiol layers. The organothiols are not epitaxially ordered with respect to the muscovite mica lattice, in contrast to those grown on gold substrates. The thiol layers are found to have similar polymorph‐selectivity for calcite growth as found for equivalent layers grown directly on gold substrates but give only limited control over the contact face.

The ultraflat organothiol layers can potentially be used in many of the applications that already exist for organothiol layers on gold, eliminating the need for gold, for example, as biosensors, protein binding, and in the fabrication of carbon nanosheets, and, additionally, for new applications that require the mobility, adaptability and healing properties of these organothiol layers.

## Conflict of interest

The authors declare no conflict of interest.

## Supporting information

As a service to our authors and readers, this journal provides supporting information supplied by the authors. Such materials are peer reviewed and may be re‐organized for online delivery, but are not copy‐edited or typeset. Technical support issues arising from supporting information (other than missing files) should be addressed to the authors.

Supplementary
