# Supplementary material for: Organothiol Monolayer Formation Directly on Muscovite Mica
Source: Angew Chem Int Ed Engl. 2019 Dec 18;59(6):2323–7. doi: 10.1002/anie.201913327 (PMC7003791; doi:10.1002/anie.201913327)
Supplement: Supplementary file 1 — Supplementary [file ANIE-59-2323-s001.pdf]

Angewandte Chemie
Eine Zeitschrift der Gesellschaft Deutscher Chemiker

Supporting Information

Organothiol Monolayer Formation Directly on Muscovite Mica

*Wester de Poel, Sander J. T. Brugman, Kim H. A. van de Ven, Anouk Gasseling, Jordi de Lange, Eleanor R. Townsend, Anthonius H. J. Engwerda, Maciej Jankowski, Melian A. R. Blijlevens, Ben L. Werkhoven, Jakub Drnec, Francesco Carlà, Roberto Felici, Aashish Tuladhar, Narendra M. Adhikari, James J. De Yoreo, Johannes A. A. W. Elemans, Willem J. P. van Enckevort, Alan E. Rowan, and Elias Vlieg**

anie_201913327_sm_miscellaneous_information.pdf

SI-1 Additional AFM measurements of different organothiol molecular layers on K-terminated muscovite mica

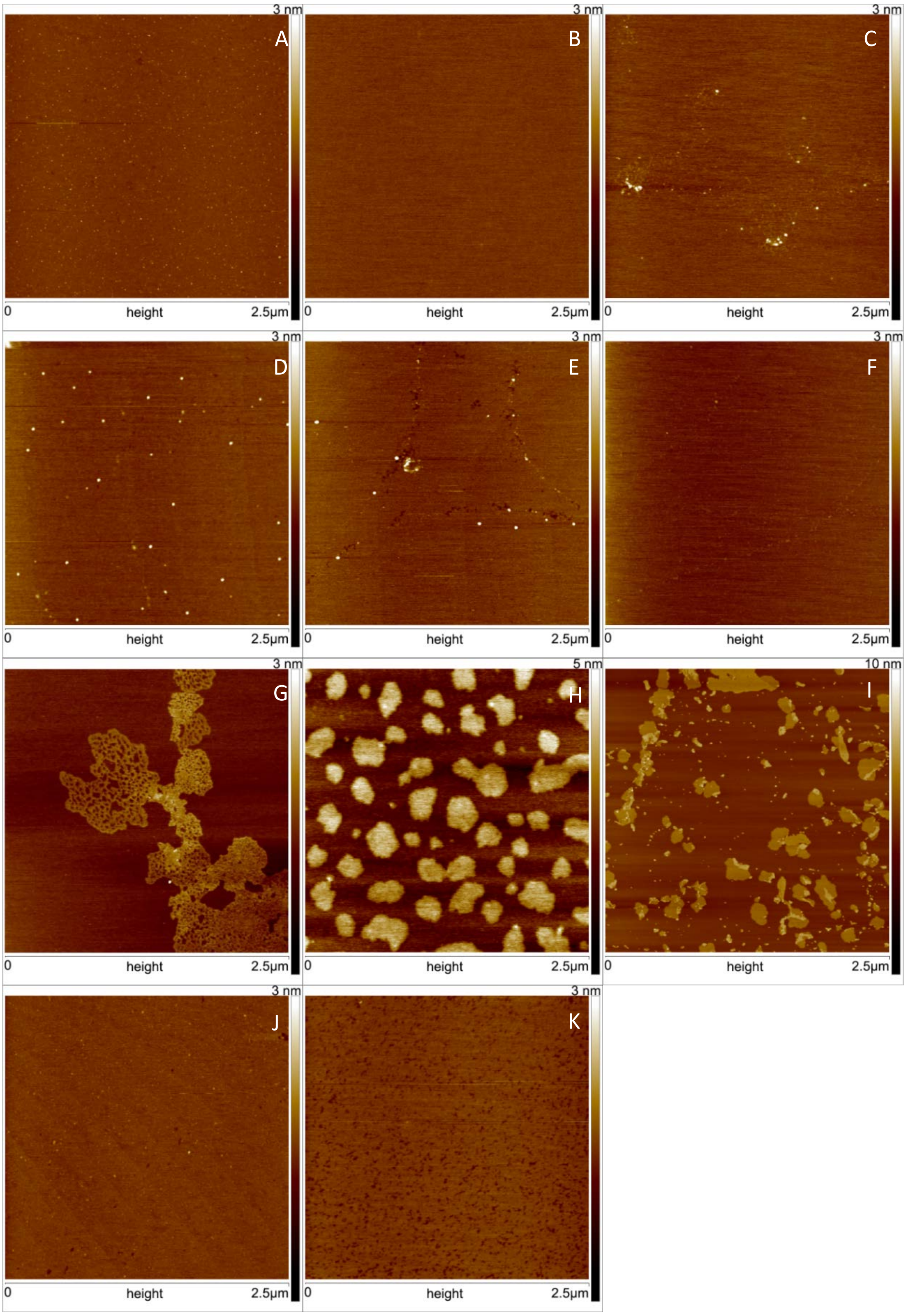

Figure S1 Atomic force microscopy height images of different organothiol layers on potassium-terminated muscovite mica, 1-hexadecanethiol (A), 1-octanethiol (B), L-cysteine (C), 11-mercapto-1-undecanol (D), 6-mercapto-1-hexanol (E),1-undecanethiol (F), 1-octadecanethiol (G), 1,8-octanedithiol (H), 16-mercaptohexanoic acid (I), 6-mercaptohexanoic acid (J), and 4-biphenylthiol (K).

SI-2 Illustration of height measurement and mobility estimate by nanoshaving using contact mode AFM

A nanoshaving experiment was conducted as follows; the layer of 1-hexadecanethiol (or other organothiol) molecules was scraped away in contact mode AFM over an area of 2.5 by 2.5 μm^2, after which a larger area of 10 by 10 μm^2 was scanned continuously, while exerting a lower pressure to avoid nanoshaving of the molecules in this larger area (Figure S2). A square depression is visible in the layer in Figure S2A, the depth of which corresponds to a single molecular layer thickness (0.7 nm). The nanoshaved area is filled up again after two hours of scanning, which points to (limited) molecular mobility of the layer in contact with the muscovite mica surface. The tip may also have contributed to restoring the closed layer.

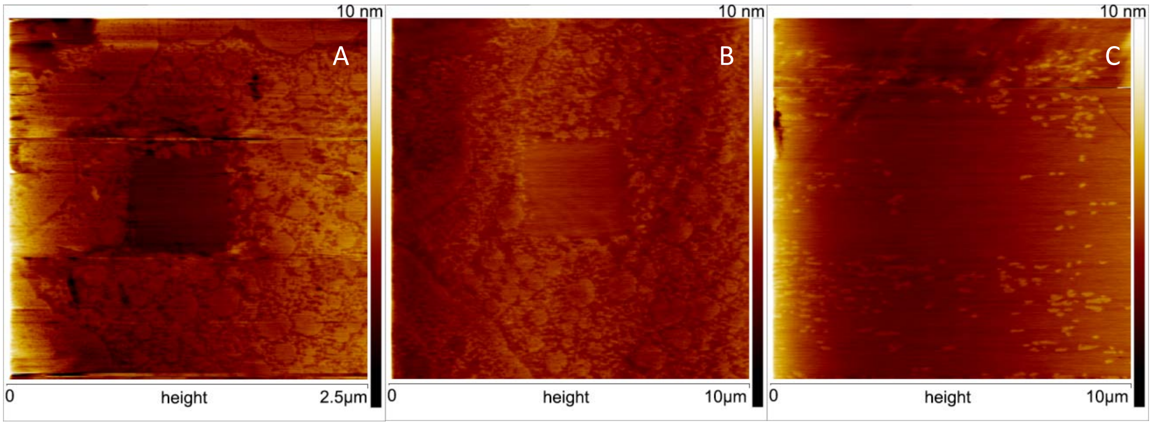

Figure S2 AFM height images of 1-hexadecanethiol on potassium-terminated muscovite mica, first measurement (A), the same area after 63 minutes (B) and 126 minutes (C) of scanning.

SI-3 Surface X-ray diffraction data sets

The measured surface X-ray diffraction data for 6-mercaptohexanoic acid on K-terminated muscovite mica is shown in Figure S3. The blue line depicts a fit for only K-terminated muscovite mica, which is sufficient to explain all crystal truncation rods measured, except for the specular. This shows that the molecules do not have an epitaxial relationship with muscovite mica. To correctly explain the specular data a model is required that includes a layer of 6-mercaptohexanoic acid, which is shown in red in Figure S3. The thiol layer on top of the muscovite mica surface was modelled in two ways: with two oriented 6-mercaptohexanoic acid molecules (red line Figure S4), or with a generic electron density profile (black line Figure S4). The z-projected electron density derived from these fits are shown in Figure S4. The electron density profile is similar in both cases, and the layer thickness is consistent (1 nm). The same fit for the specular data is obtained in both cases, without significant differences. From this, we can derive that there is a closed molecular layer consisting of 2.6 molecules of 6-mercaptohexanoic acid per surface unit cell of muscovite mica, ordered in two layers, where the bottom layer has a higher density than the top layer.

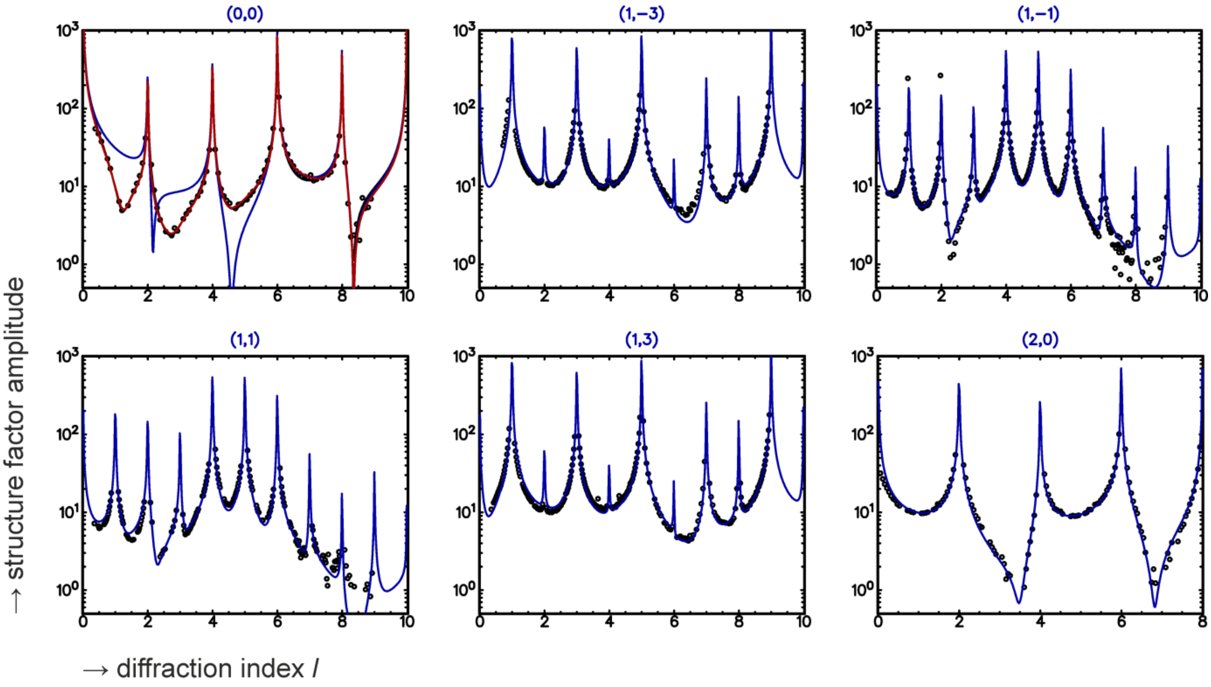

Figure S3 SXRD data (black dots) for 6-mercaptohexanoic acid on K-terminated muscovite mica. Blue line: fit based on bare K-terminated muscovite mica, red line: fit based on K-terminated muscovite mica with a layer of 6-mercaptohexanoic acid. The crystallographic *l*-direction is depicted on the horizontal axis, and the structure factor amplitude is depicted on the vertical axis. The labels above each graph indicate the *h* and *k*-values for the specific crystal truncation rod.

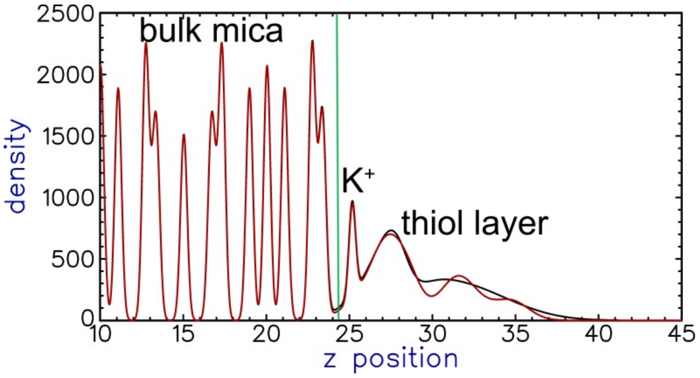

Figure S4 Z-projected electron density (electrons per unit cell) as function of height (in Å) from the SXRD dataset of K-terminated muscovite mica with a layer of 6-mercaptohexanoic acid. Black line: z-projected electron density derived from a generic model, red line: z-projected electron density derived from a model with molecules.

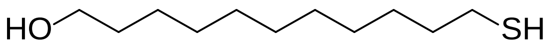

11-mercapto-1-undecanol

For the remaining datasets we only show the specular fit, while the whole dataset was used to arrive at the presented results. The obtained SXRD data of 11-mercapto-1-undecanol on potassium-terminated muscovite mica required 2 molecules in the model in order to obtain a good fit. The lowest lying molecule has an occupancy of 0.93 molecules per unit cell, and the highest lying molecule 0.64 molecules per unit cell. The height of these molecules (0.7 nm) corresponds well with the measured value obtained with AFM.

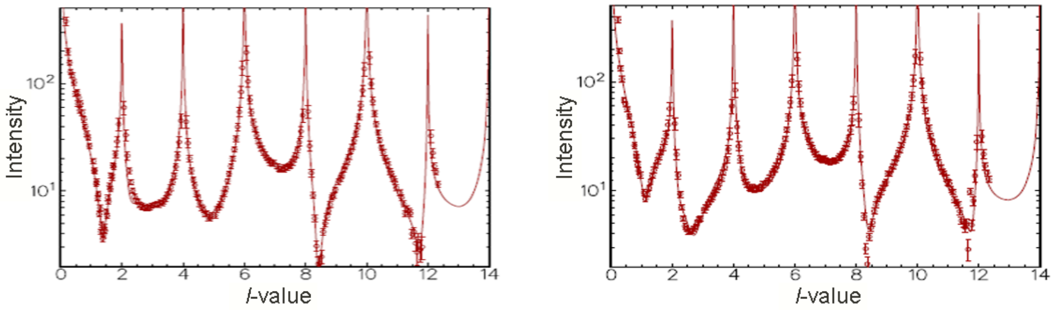

Figure S5 SXRD specular data of 11-mercapto-1-undecanol on K-terminated (left), and Cu-terminated (right) muscovite mica (dots), and fit (line). The y-axis depicts the structure factor amplitude and the x-axis depicts the l-value.

The obtained SXRD data of 11-mercapto-1-undecanol on copper-terminated muscovite mica required 2 molecules in the model in order to obtain a good fit. The lowest lying molecule has an occupancy of 0.32 molecules per unit cell, and the highest lying molecule 1.69 molecules per unit cell. The combined height of these layers (1.0 nm) is 0.7 nm lower than the measured value obtained with AFM.

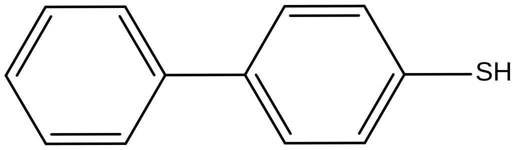

4-biphenylthiol

The obtained SXRD data of 4-biphenylthiol on potassium-terminated muscovite mica required 2 molecules in the model in order to obtain a good fit. The lowest lying molecule has an occupancy of 1.46 molecules per unit cell, and the highest lying molecule 1.04 molecules per unit cell. The combined height of these layers (1.0 nm) is 0.5 nm higher than the measured value obtained with AFM.

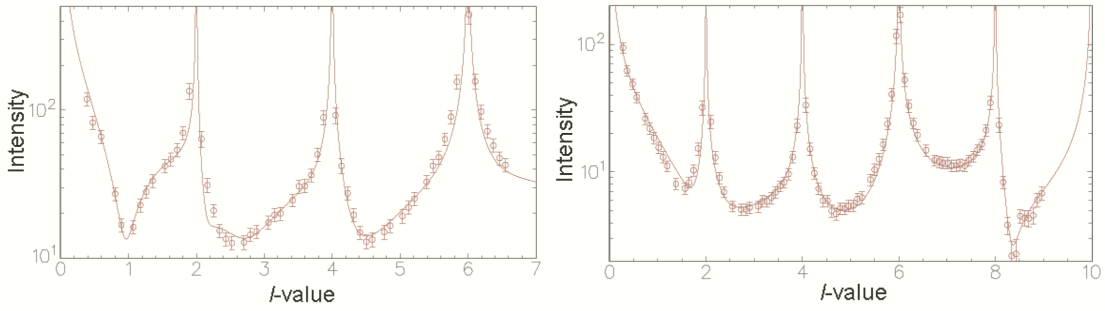

Figure S6 SXRD specular data of 4-biphenylthiol on K-terminated (left), and Cu-terminated (right) muscovite mica (dots), and fit (line). The y-axis depicts the structure factor amplitude and the x-axis depicts the l-value.

The obtained SXRD data of 4-biphenylthiol on copper-terminated muscovite mica required 1 molecule in the model in order to obtain a good fit. The molecule has an occupancy of 0.99 molecules per unit cell. The height of the molecule (0.3 nm) corresponds well with the measured value obtained with AFM.

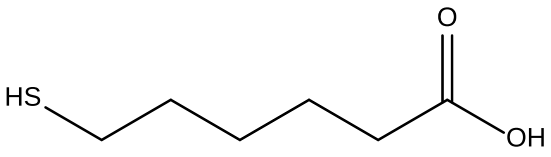

6-mercaptohexanoic acid

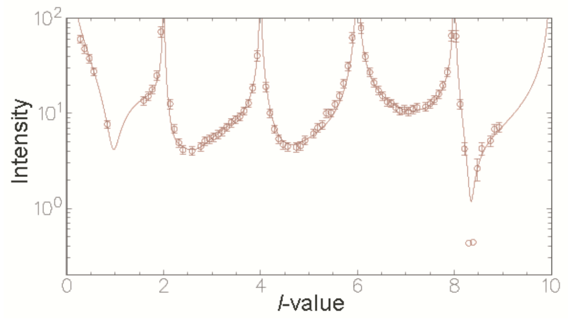

Figure S7 SXRD specular data of 6-mercaptohexanoic acid on Cu-terminated muscovite mica (dots), and fit (line). The y-axis depicts the structure factor amplitude and the x-axis depicts the l-value.

The obtained SXRD data of 6-mercaptohexanoic acid on copper-terminated muscovite mica required 2 molecules in the model in order to obtain a good fit. The lowest lying molecule has an occupancy of 1.53 molecules per unit cell, and the highest lying molecule 1.34 molecules per unit cell. The combined height of these layers (1.0 nm) is 0.9 nm lower than the measured value obtained with AFM.

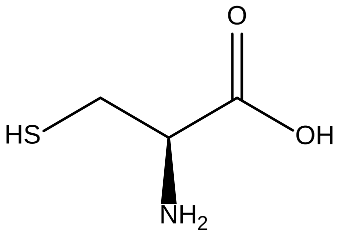

l-cysteine

The obtained SXRD data of L-cysteine on potassium-terminated muscovite mica required 2 molecules in the model in order to obtain a good fit. The lowest lying molecule has an occupancy of 1.92 molecules per unit cell, and the highest lying molecule 1.74 molecules per unit cell. The combined height of these layers (0.9 nm) corresponds well with the measured value obtained with AFM.

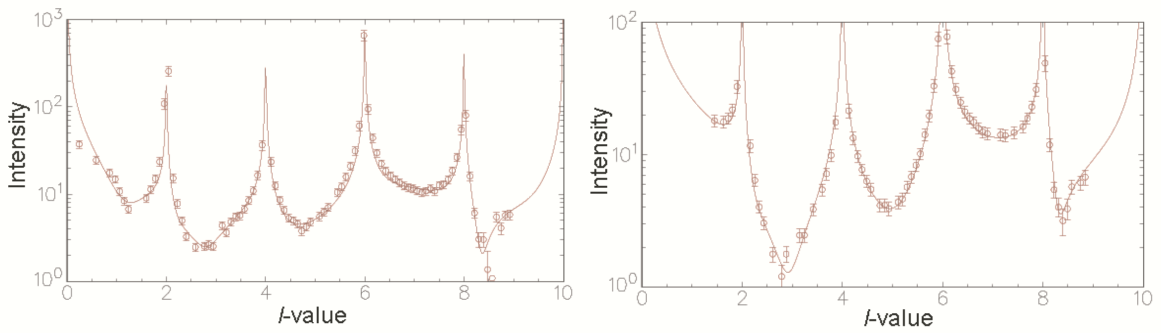

Figure S8 SXRD specular data of L-cysteine on K-terminated (left), and Cu-terminated (right) muscovite mica (dots), and fit (line). The y-axis depicts the structure factor amplitude and the x-axis depicts the l-value.

The obtained SXRD data of L-cysteine on copper-terminated muscovite mica required 1 molecule in the model in order to obtain a good fit. The molecule has an occupancy of 1.03 molecules per unit cell. The height of the molecule (0.3 nm) corresponds well with the measured value obtained with AFM.

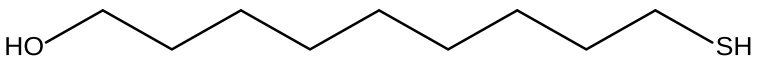

9-mercapto-1-nonanol

The obtained SXRD data of 9-mercapto-1-nonanol on potassium-terminated muscovite mica required 2 molecules in the model in order to obtain a good fit. The lowest lying molecule has an occupancy of 1.86 molecules per unit cell, and the highest lying molecule 0.25 molecules per unit cell. The combined height of these layers (1.2 nm) is 0.5 nm higher than the measured value obtained with AFM.

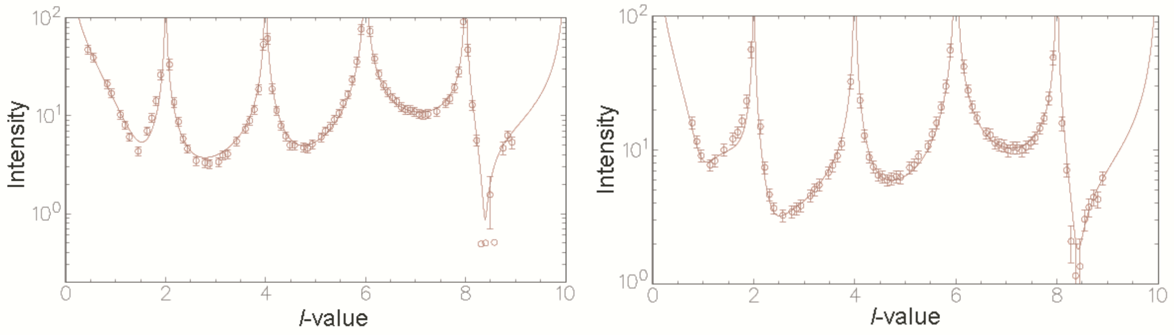

Figure S9 SXRD specular data of 9-mercapto-1-nonanol on K-terminated (left), and Cu-terminated (right) muscovite mica (dots), and fit (line). The y-axis depicts the structure factor amplitude and the x-axis depicts the l-value.

The obtained SXRD data of 9-mercapto-1-nonanol on copper-terminated muscovite mica required 2 molecules in the model in order to obtain a good fit. The lowest lying molecule has an occupancy of 1.67 molecules per unit cell, and the highest lying molecule 0.66 molecules per unit cell. The combined height of these layers (1.3 nm) corresponds well with the measured value obtained with AFM.

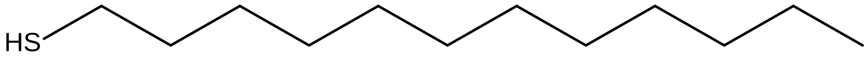

1-dodecanethiol

The obtained SXRD data of 1-dodecanethiol on potassium-terminated muscovite mica required 2 molecules in the model in order to obtain a good fit. The lowest lying molecule has an occupancy of 1.35 molecules per unit cell, and the highest lying molecule 1.35 molecules per unit cell. The combined height of these layers (0.8 nm) is 0.7 nm lower than the measured value obtained with AFM.

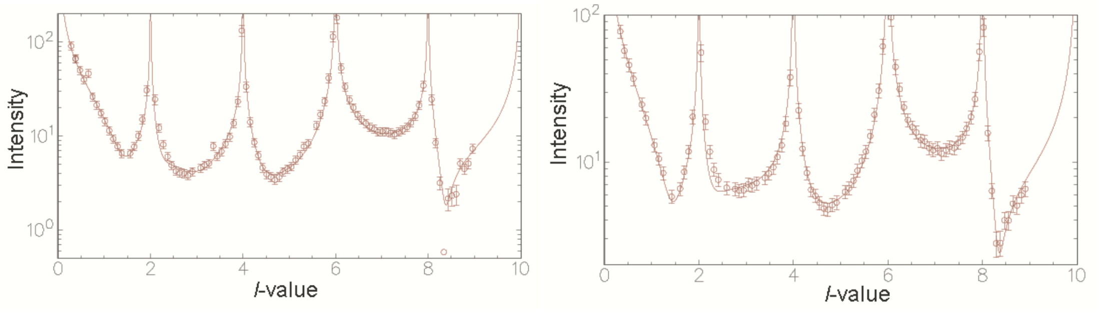

Figure S10 SXRD specular data of 1-dodecanethiol on K-terminated (left), and Cu-terminated (right) muscovite mica (dots), and fit (line). The y-axis depicts the structure factor amplitude and the x-axis depicts the l-value.

The obtained SXRD data of 1-dodecanethiol on copper-terminated muscovite mica required 1 molecule in the model in order to obtain a good fit. The molecule has an occupancy of 1.31 molecules per unit cell. The height of the molecule (0.6 nm) is 0.6 nm lower than the measured value obtained with AFM.

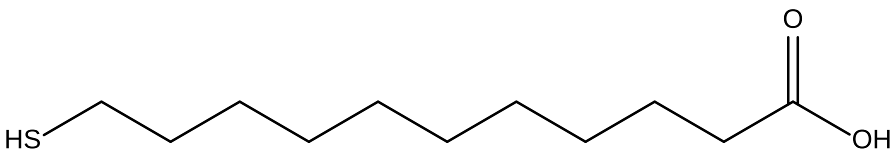

11-mercaptoundecanoic acid

The obtained SXRD data of 11-mercaptoundecanoic acid on potassium-terminated muscovite mica required 2 molecules in the model in order to obtain a good fit. The lowest lying molecule has an occupancy of 1.13 molecules per unit cell, and the highest lying molecule 0.90 molecules per unit cell. The combined height of these layers (0.7 nm) corresponds well with the measured value obtained with AFM.

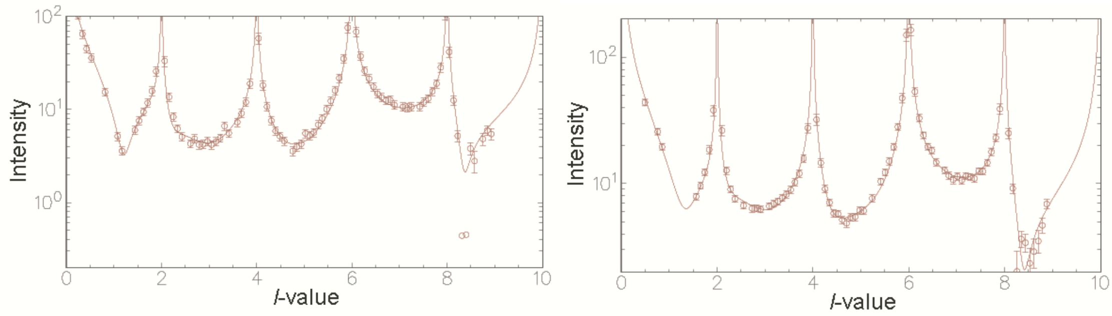

Figure S11 SXRD specular data of 11-mercaptoundecanoic acid on K-terminated (left), and Cu-terminated (right) muscovite mica (dots), and fit (line). The y-axis depicts the structure factor amplitude and the x-axis depicts the l-value.

The obtained SXRD data of 11-mercaptoundecanoic acid on copper-terminated muscovite mica required 2 molecules in the model in order to obtain a good fit. The lowest lying molecule has an occupancy of 1.73 molecules per unit cell, and the highest lying molecule 0.49 molecules per unit cell. The combined height of these layers (0.7 nm) is 1.1 nm lower than the measured value obtained with AFM.

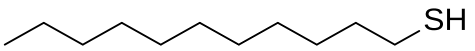

1-undecanethiol

The obtained SXRD data of 1-undecanethiol on potassium-terminated muscovite mica required 2 molecules in the model in order to obtain a good fit. The lowest lying molecule has an occupancy of 0.90 molecules per unit cell, and the highest lying molecule 0.21 molecules per unit cell. The combined height of these layers (1.6 nm) is 1.0 nm higher than the measured value obtained with AFM and possibly corresponds to a bilayer.

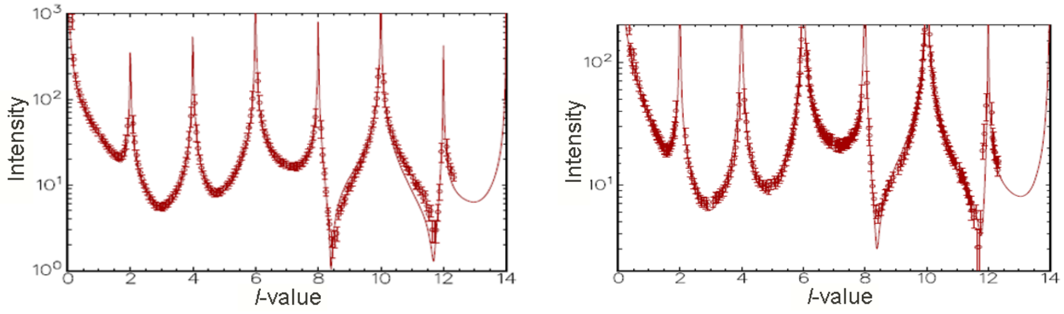

Figure S12 SXRD specular data of 1-undecanethiol on K-terminated (left), and Cu-terminated (right) muscovite mica (dots), and fit (line). The y-axis depicts the structure factor amplitude and the x-axis depicts the l-value.

The obtained SXRD data of 1-undecanethiol acid on copper-terminated muscovite mica required 2 molecules in the model in order to obtain a good fit. The lowest lying molecule has an occupancy of 1.13 molecules per unit cell, and the highest lying molecule 0.37 molecules per unit cell. The combined height of these layers (1.0 nm) is 0.5 nm higher than the measured value obtained with AFM and might also correspond to a bilayer.

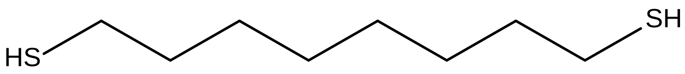

1,8-octanedithiol

The obtained SXRD data of 1,8-octanedithiol on potassium-terminated muscovite mica required 2 molecules in the model in order to obtain a good fit. The lowest lying molecule has an occupancy of 1.14 molecules per unit cell, and the highest lying molecule 1.22 molecules per unit cell. The combined height of these layers (1.2 nm) is 1.1 nm lower than the measured value obtained with AFM.

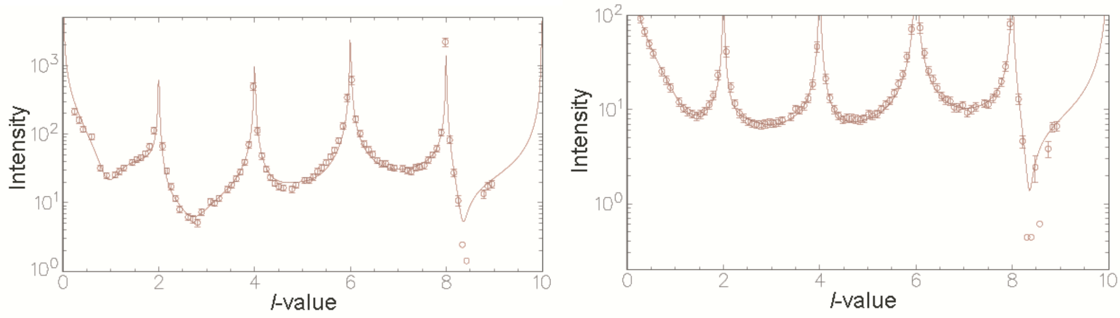

Figure S13 SXRD specular data of 1,8-octanedithiol on K-terminated (left), and Cu-terminated (right) muscovite mica (dots), and fit (line). The y-axis depicts the structure factor amplitude and the x-axis depicts the l-value.

The obtained SXRD data of 1,8-octanedithiol on copper-terminated muscovite mica required 2 molecules in the model in order to obtain a good fit. The lowest lying molecule has an occupancy of 1.64 molecules per unit cell, and the highest lying molecule 0.63 molecules per unit cell. The combined height of these layers (1.0 nm) corresponds well with the measured value obtained with AFM.

SI-4 Summary table

Table S1 Information about the height of the layers as obtained from AFM and SXRD, an asterisk indicates good agreement of SXRD with AFM data

Molecule	Termination	(AFM) Height 1st layer (nm)	(AFM) Height 2nd layer (nm)	Minimum number of molecules required to explain the AFM height	(SXRD) Number of molecules per unit cell	(SXRD) Layer thickness (nm)
**16-mercapto-hexadecanoic acid**	K	1.1 ± 0.5	2.9 ± 0.5	1, 2		
**16-mercapto-hexadecanoic acid**	Cu	1.0 ± 0.5	2.6 ± 0.5	1, 2		
**L-cysteine***	K	0.6 ± 0.3		1	3.7	0.9 ± 0.1
**L-cysteine***	Cu	0.5 ± 0.3		1	1.0	0.3 ± 0.1
**11-mercapto-1-undecanol**	K	0.6 ± 0.2		1	1.6	0.7 ± 0.1
**11-mercapto-1-undecanol**	Cu	1.7 ± 0.5		1	2.0	1.0 ± 0.1
**9-mercapto-1-nonanol**	K	0.7 ± 0.2		1	2.1	1.2 ± 0.1
**9-mercapto-1-nonanol***	Cu	1.3 ± 0.5		1	2.3	1.3 ± 0.1
**6-mercapto-hexanol**	K	0.6 ± 0.2		1		
**6-mercapto-hexanol**	Cu	2.4 ± 0.5		2		
**6-mercapto-hexanoic acid***	K	0.4 ± 0.2		1	2.6	1.0 ± 0.1
**6-mercapto-hexanoic acid**	Cu	1.9 ± 0.5		2	2.9	1.0 ± 0.1
**1-octanethiol**	K	0.5 ± 0.2		1		
**1-octanethiol**	Cu	0.5 ± 0.2		1		
**1,8-octanedithiol**	K	2.3 ± 0.7		2	2.4	1.2 ± 0.1
**1,8-octanedithiol***	Cu	1.7 ± 0.8		2	2.3	1.0 ± 0.1
**1-dodecanethiol**	K	1.5 ± 0.2		1	2.7	0.8 ± 0.1

**1-dodecanethiol**	Cu	1.2 ± 0.2		1	1.3	0.6 ± 0.1
**1-hexadecanethiol**	K	0.7 ± 0.4		1		
**1-hexadecanethiol**	Cu	0.9 ± 0.3	5.5 ± 2.5	1, 3		
**11-mercapto-undecanoic acid***	K	0.5 ± 0.2		1	2.0	0.7 ± 0.1
**11-mercapto-undecanoic acid**	Cu	1.8 ± 0.6		1	2.2	0.7 ± 0.1
**4-biphenylthiol**	K	0.5 ± 0.2		1	2.5	1.0 ± 0.1
**4-biphenylthiol***	Cu	0.3 ± 0.2		1	1.0	0.3 ± 0.1
**1-undecanethiol**	K	0.6 ± 0.2		1	1.1	1.6 ± 0.1
**1-undecanethiol**	Cu	0.5 ± 0.2		1	1.5	1.0 ± 0.1
**1-octadecanethiol**	K	0.6 ± 0.2	1.1 ± 0.2	1, 1		
**1-octadecanethiol**	Cu	0.5 ± 0.2	1.0 ± 0.2	1, 1		

SI-5 AFM measurements regarding the stability of the organothiol layers at ambient conditions

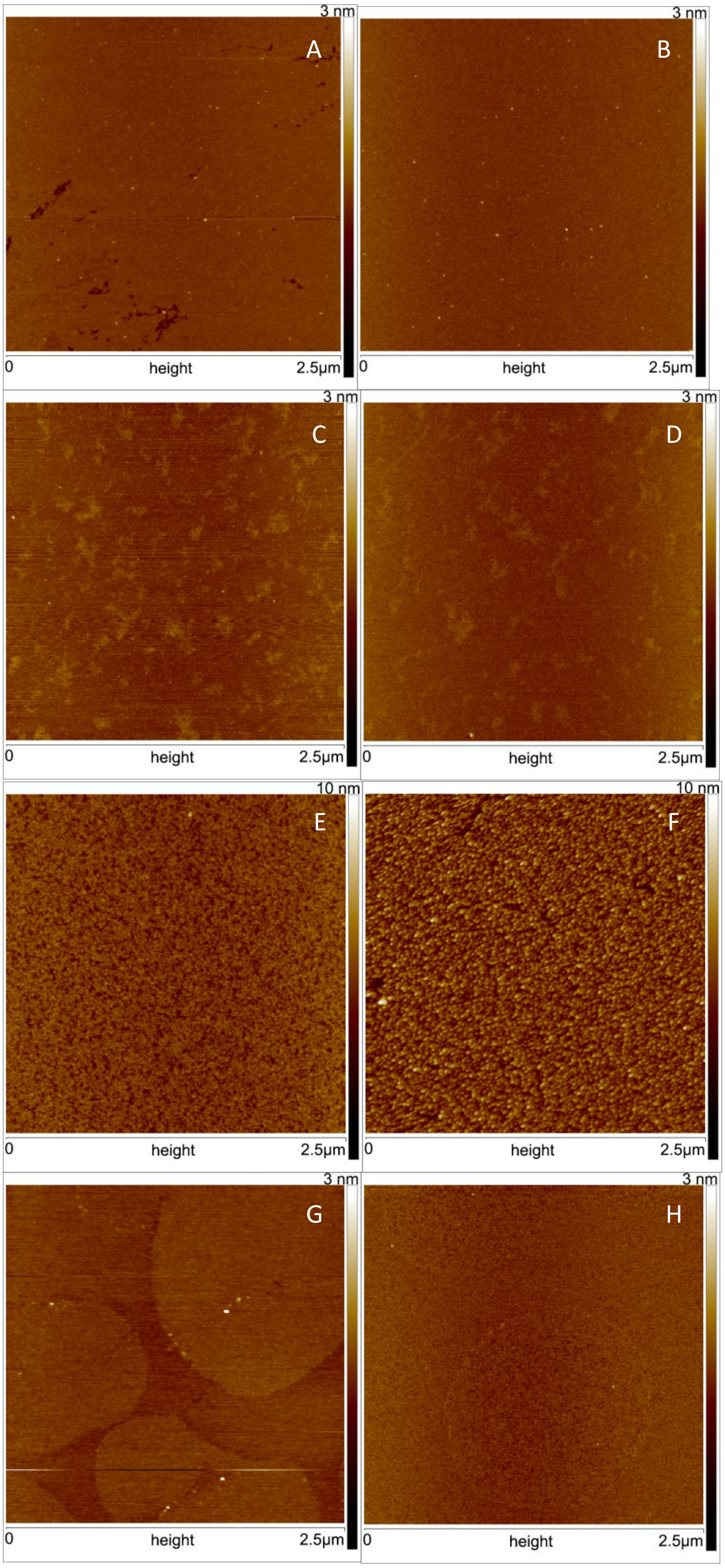

Figure S14 Layers of 9-mercapto-1-nonanol (A), L-cysteine (C) on K-terminated muscovite mica on the day of fabrication, and 6 days later (B,D), and layers of 1,8-octanedithiol on Cu-terminated muscovite mica (E), and 4-biphenylthiol on K-terminated muscovite mica (G) on the day of fabrication, and 6 days later (F,H). Measurements were conducted at different areas of the sample.

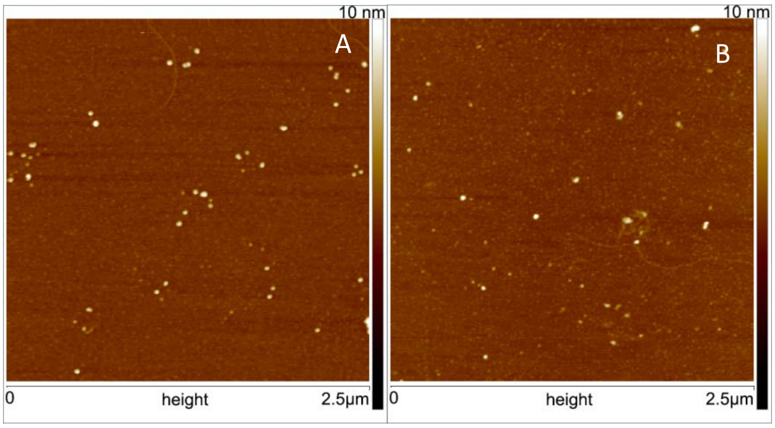

Figure S15 16-mercaptohexadecanoic acid (A), and L-cysteine (B) layer on Cu-terminated muscovite mica, after being immersed in water for 90 hours.

SI-6 AFM measurement of two different mobile partial organothiol layers

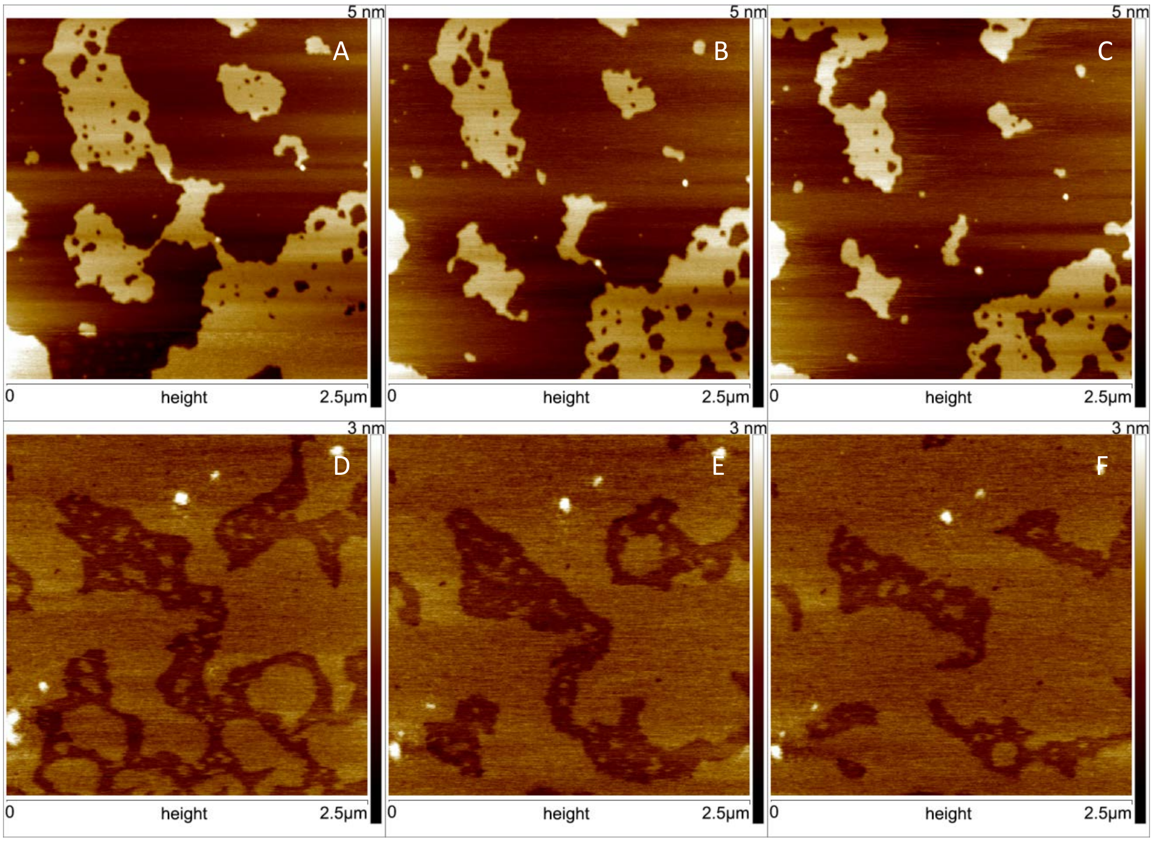

Figure S16 Consecutive AFM height images of 9-mercapto-1-nonanol (A-C), and 1,8-octanedithiol (D-F) on potassium-terminated muscovite mica. The elapsed time between two images is 9 minutes.

SI-7 AFM measurements of different organothiol molecular layers on Cu-terminated muscovite mica

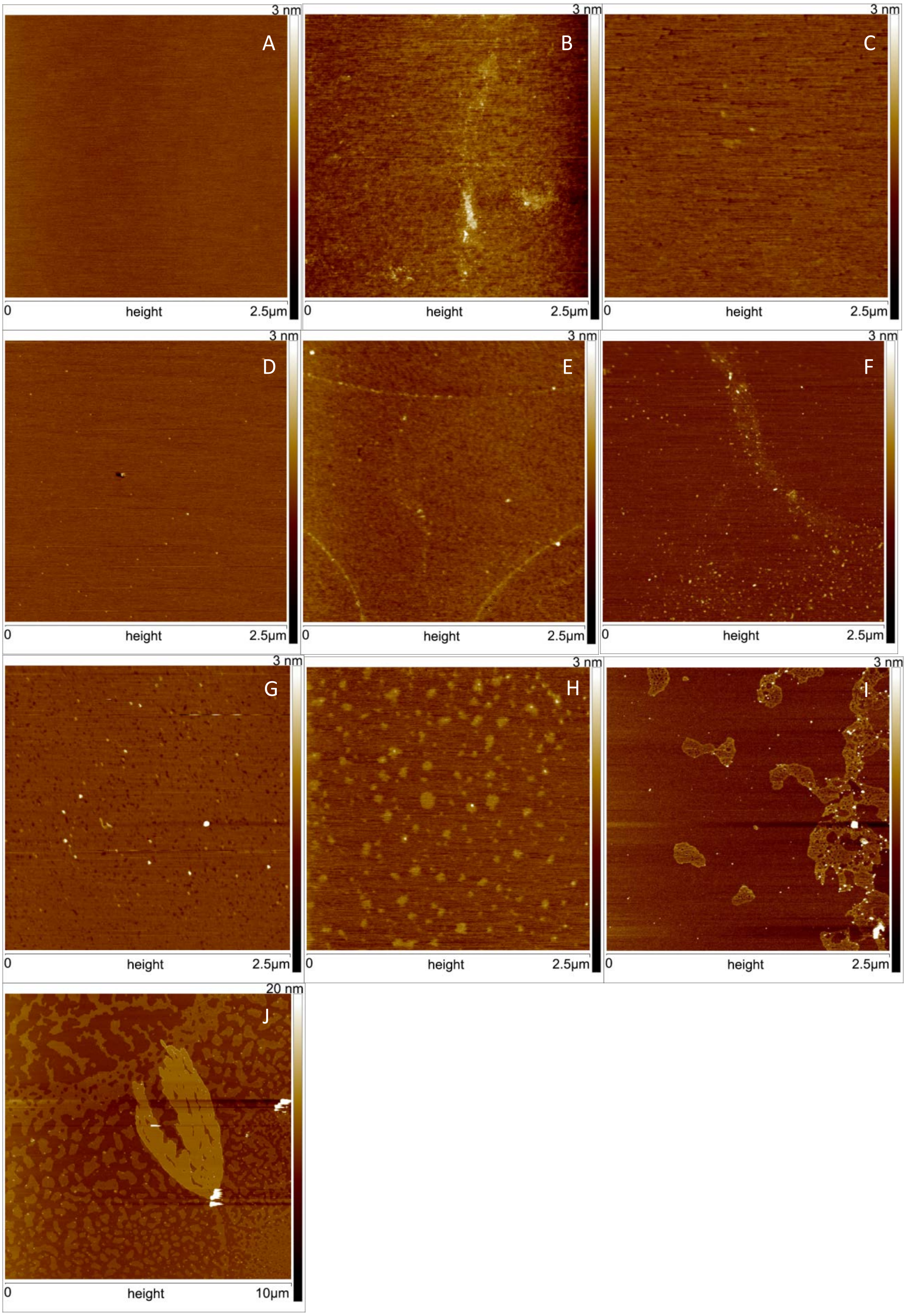

Figure S17 AFM height images of Cu-terminated muscovite mica (A), containing 4-biphenylthiol (B), 1-hexadecanethiol (C), 1-dodecanethiol (D), 9-mercapto-1-nonanol (E), 1-undecanethiol (F), L-cysteine (G), 6-mercaptohexanoic acid (H), 1-octadecanethiol (I), and 1-hexadecanethiol.

SI-8 Nanoshaving on a Cu-terminated muscovite mica surface

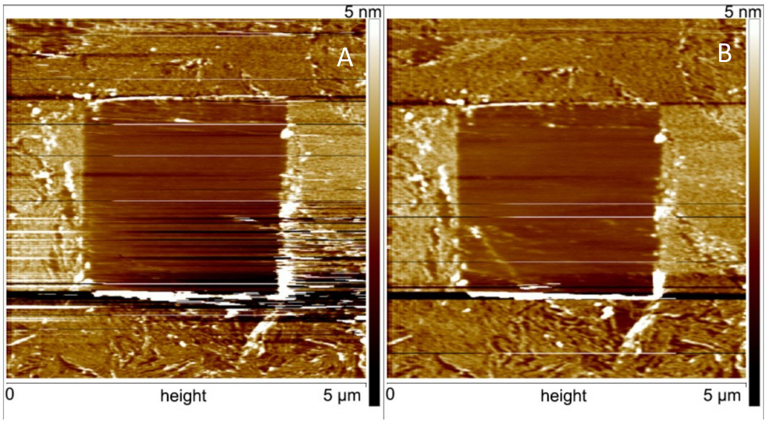

Figure S18 AFM height images of Cu-terminated muscovite mica with 6-mercaptohexanoic acid, (A) measured directly after scraping, (B) the same surface area after 3 more scans, 10 minutes later, the depression is virtually unchanged. The high scanning speed is responsible for the emergence of stripes and the elongation of some small features.

SI-9 Comparison epitaxial calcite

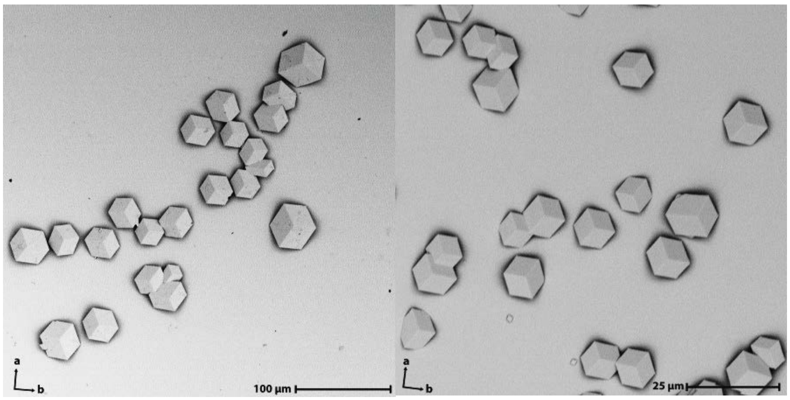

Figure S19 Scanning electron microscopy images of an area with epitaxial calcite growth on a 11-mercapto-1-undecanol functionalized Cu-terminated muscovite mica surface (left) and epitaxial calcite crystals grown on muscovite mica weathered at 43% relative humidity for 1.5 h [1](right) showing the same (006) orientation parallel to the mica basal plane in both images. The muscovite lattice vectors are indicated in the lower left corner of the figures and show that the calcite crystals in both images have the same epitaxial relationship with the underlying muscovite mica crystal.

Table S2 Calcium carbonate crystallization data on various substrates. These values are averages from 5 samples from at least two crystallization batches

Substrate	crystals per mm	% calcite	% calcite(006)	size calcite (µm)
K-mica freshly cleaved (reference)	108 ± 127	67 ± 29	15 ± 4	32 ± 8
K-mica + 11-mercapto-1-undecanol	52 ± 38	93 ± 2	30 ± 7	27 ± 4
Cu-mica + 11-mercapto-1-undecanol	24 ± 5	94 ± 3	38 ± 11	27 ± 3
K-mica + 11-mercaptoundecanoic acid	68 ± 14	99 ± 1	15 ± 3	17 ± 4
Cu-mica + 11-mercaptoundecanoic acid	99 ± 44	99 ± 1	12 ± 1	13 ± 2
K-mica + 1-undecanethiol	142 ± 151	61 ± 1	27 ± 2	24 ± 9
Cu-mica + 1-undecanethiol	32 ± 8	74 ± 19	37 ± 10	28 ± 2

SI-10 Vibrational Sum-Frequency Generation IR spectroscopy measurements

A picosecond scanning vSFG spectrometer (EKSPLA, Lithuania) and Bruker Vertex 70 Hyperion 1000 spectrometer (Bruker, Germany) were used to perform vSFG IR (interface-specific) and FTIR (bulk) measurements, respectively. Briefly, the vSFG spectrometer is a commercial setup that uses 532.1 nm visible beam and a tunable infra-red (IR) beam overlapped spatially and temporally at the sample surface. The angle of incidence is 65° and 55° for the visible and the IR beam, respectively. The spatial resolution of the setup is ~6 cm^{-1}. An SSP polarization geometry (where S, S, and P refer to the polarization of sum frequency, visible, and IR photons, respectively) was used. The vSFG measurements were conducted on organothiol covered mica samples prepared as discussed in SI-12. For the FTIR measurements, a drop of 1-undecanethiol and powder of a grain of 11-mercapto-1-undecanol were spread onto IR transparent clean microscope slides. The FTIR spectra were recorded in a transmission mode with an average of 64 scans with a resolution of 4 cm^{-1}.

vSFG IR spectroscopy has been extensively used to quantitatively analyse molecular orientation and chain conformation of organic monolayers at interfaces in a SSP polarization geometry [2-4]. This is done by comparing the relative intensities of the CH_3 symmetric stretch (ss) at 2880 cm^{-1} and the CH_2 (ss) at 2850 cm^{-1}. P-polarized light probes only vibrations with IR transition moment aligned primarily along the surface normal. For a tightly packed monolayer with upright alkyl chains, a large signal from CH_3(ss) and a weak or non-existent signal from CH_2(ss) is expected. This is because the transition moment of CH_2 groups in a highly ordered monolayer will be aligned in the surface plane and hence not excited by the P-polarized IR light. In addition, a local inversion symmetry is found for the CH_2 groups in all-trans chains, which makes it SFG inactive. In contrast, for organic monolayers with low surface coverage and/or gauche defects, the CH_3 (ss) signal is expected to be reduced and CH_2(ss) signal should increase.

The vSFG spectroscopy was performed for two representative organothiols: 1-undecanethiol and 11-mercapto-1-undecanol. The C-H stretching vibration of the data is shown in figure S20 for K-terminated mica and figure S21 for Cu-terminated mica. On clean mica, there are no CH-related peaks, but for the two SAMs the signal is very clear. This is additional proof for the presence of the organothiol layers. The strong symmetric stretching (ss) peak for CH_3 at 2880 cm^{-1} shows that this group is pointing away from the mica, and thus 1-undecanethiol is bonded to the mica surface through the thiol group. (11-mercapto-1-undecanol does not have the CH_3 end group, and thus for this molecule such a peak is absent). Also for 11-mercapto-1-undecanol the bond to mica is through the thiol group, because the SH peak that is clearly visible in bulk material (figure S22), but is absent for the SAM of the molecule on mica (figure S23), which means that the thiol group is in contact with the mica.

If the SAMs were highly ordered, as explained in the previous paragraph, one would expect the signal due to CH_2(ss) to be very small or non-existent. The fact that these peaks are observed thus means that the SAMs are quite disordered (in agreement with the XRD results). For 1-undecanethiol we have both CH_2 and CH_3 peaks and the ratio CH_3(ss):CH_2(ss) is close to 1, indicating that the SAM is disordered. For the Cu-terminated mica this ratio is much lower than for K-terminated mica, pointing to an increased disorder in the layer. From stability experiments using AFM, we know that the layers on Cu-terminated mica are more stable, i.e. the bond to the mica is stronger. This apparently does not lead to a higher order within the layer.

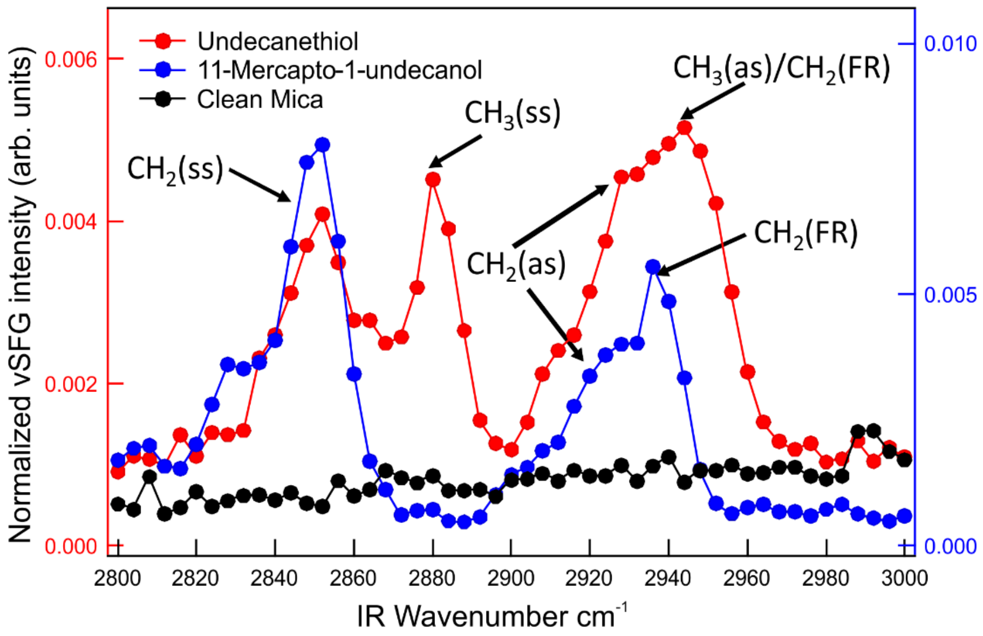

Figure S20 vSFG data on K-terminated mica with the peaks from the different symmetric (ss), asymmetric stretching (as) modes and Fermi resonance (FR) assigned.

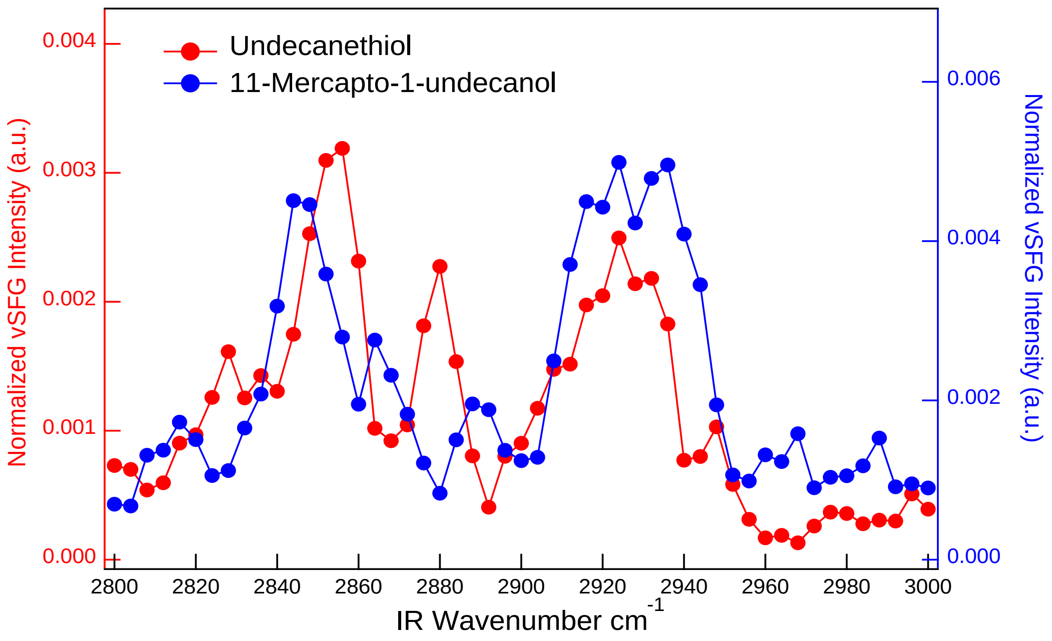

Figure S21 vSFG data on Cu-terminated mica.

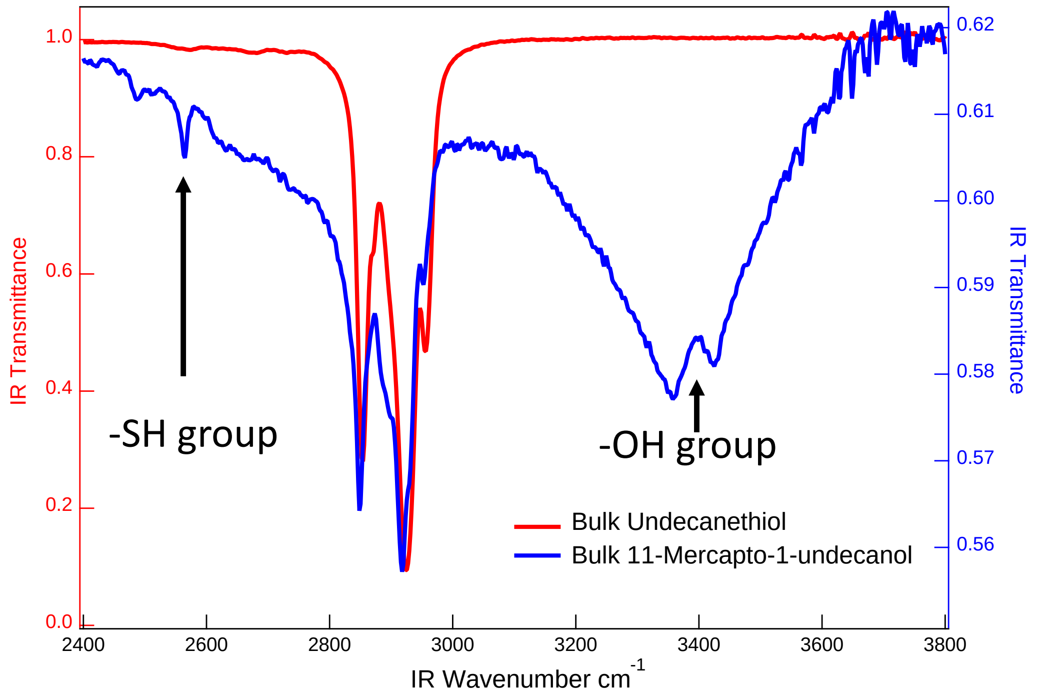

Figure S22 FTIR spectra of the two organothiols in bulk, showing the location of the different peaks.

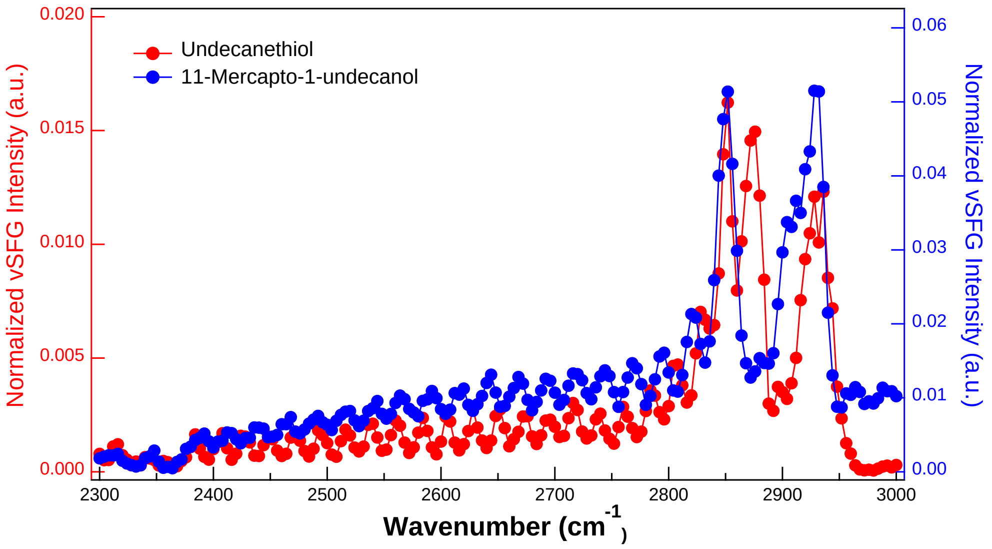

Figure S23 vSFG data on K-terminated mica covering a wider spectral range, including the location where the S-H stretching peak is expected. The wiggles in the data are an artifact due to optical fringes caused by the fact that the mica substrate used for the experiment was very thin.

SI-11 Contact angle measurements

In order to obtain additional proof for the presence of a monolayer, contact angle measurements were performed of water in contact with clean mica and with a number of organothiols. In all cases, the angle with organothiol film is much larger, proving the chemical modification of the substrate by the application of the corresponding SAM. The error in the measured angle is ±5°.

**system**	**termination**	**contact angle (°)**
clean mica	K	0
clean mica	Cu	0
6-mercaptohexanoic acid	K	19
6-mercaptohexanoic acid	Cu	17
6-mercapto-1-hexanol	K	19
6-mercapto-1-hexanol	Cu	28
9-mercapto-1-nonanol	K	20
9-mercapto-1-nonanol	Cu	18
11-mercapto-1-undecanol	K	19
11-mercapto-1-undecanol	Cu	18
L-cysteine	K	25
L-cysteine	Cu	35
16-mercaptohexadecanoic acid	K	29

SI-12 Experimental details

Materials

Muscovite mica (quality grade ASTM-V1, a = 0.51906 nm, b = 0.9008 nm, c = 2.0047 nm, α = γ = 90°, β = 95.757°, space group *C 2/c*, chemical formula $KAl_2(Si_3Al)O_{10}(OH)_2$) was obtained from S&J Trading Inc. (Glen Oaks, NY, USA). 1-octanethiol (98.5% purity), 1,8-octanedithiol (97% purity), 1-dodecanethiol (98% purity), 1-hexadecanethiol (96% purity), 11-mercaptoundecanoic acid (95% purity), L-cysteine (97% purity), 11-mercapto-1-undecanol (97% purity), 9-mercapto-1-nonanol (96% purity), 6-mercaptohexanoic acid (90% purity), 1,1'-biphenyl-4-thiol (97% purity), 6-mercapto-1-hexanol (97% purity), 1-undecanethiol (98% purity), 1-octadecanethiol (>95% purity) and 16-mercaptohexadecanoic acid (92% purity) were obtained from Sigma Aldrich. Calcium chloride dihydrate (>99.5% purity) and copper chloride (99% purity) were obtained from Merck. All materials were used without further purification.

Surface preparation

Freshly cleaved muscovite mica was submerged into approximately 15 mL of a solution of 10^{-2}M organothiol in dichloromethane ≥99.8% pure (CHROMASOLV for HPLC ≥99.8% pure, obtained from Sigma Aldrich). This led to saturated solutions in the cases of L-cysteine, and 16-mercaptohexadecanoic acid. The muscovite mica was left in the solution for at least one hour, then removed from the solution and washed three times for at least 30 seconds in fresh solvent of approximately 15 mL of dichloromethane to remove the excess thiol. The sample was then dried using a gentle nitrogen gas flow for 2 minutes in a vertical position and at least one hour in a horizontal position. All experiments were performed in triplicate. The samples were characterized using AFM on the same day that they were made, and within two weeks using SXRD. The same procedure was followed for Cu-terminated muscovite mica. The ion-exchange procedure to obtain Cu-terminated muscovite mica is described elsewhere [5] and further on. Potassium-terminated muscovite mica surfaces were obtained after cleavage of the crystal along the (001) plane. Potassium-terminated muscovite mica comprises of one K^+ ion per surface unit cell (coverage of ½), while Cu^{2+}-terminated muscovite mica is expected to have only half a copper ion per surface unit cell (coverage of ¼), in order to preserve charge neutrality.

Surface characterization

AFM measurements were carried out on a Dimension 3100 AFM and a NanoScope Multimode 8 AFM with HA-NC tips for tapping mode and CSG10 tips for contact mode from NT-MDT.

SXRD was performed at beamline ID03 of the ESRF using a vertical z-axis diffractometer equipped with a 2D detector, in the stationary geometry [6]. The momentum transfer in the X-ray diffraction experiments is denoted by $\vec{Q} = h \cdot \vec{a}^* + k \cdot \vec{b}^* + l \cdot \vec{c}^*$, with $\vec{a}^*$, $\vec{b}^*$, and $\vec{c}^*$ the reciprocal lattice vectors and (hkl) the diffraction indices. The diffraction rods are oriented along the l –direction, which is perpendicular to the (001) muscovite mica cleavage surface. Most experiments were performed using a 16 keV X-ray beam having a 1 mm horizontal width and 50 μm vertical width, with an incoming angle of 0.6° for non-specular data; this led to a footprint of 1 by 5 mm^2. Measurements of 11-mercapto-1-undecanol and 1-undecanethiol were performed using a 23 keV X-ray beam having a 44 μm horizontal width and 115 μm vertical width. To prevent damage induced by the X-ray beam, a filter was added.

Structure factors from several crystal truncation rods (CTRs) were derived from the detector images using MATLAB code written for this purpose. Fitting of the SXRD data was carried out using the ROD program [7].

The surface termination was characterized prior to the full data acquisition by measuring the $(1\ 1\ 1.3)$ and $(1\ \bar{1}\ 1.3)$ reflections for a large part of the surface to make sure that measurements were carried out on a single-terminated muscovite mica surface [8]. The measurements were performed under dry conditions by placing the samples in a cell with a constant nitrogen flow.

A model was developed to fit the data and includes the bulk and a surface unit cell of muscovite mica and a specified number of thiol molecules. The fit parameters are the occupancy, location and orientation of the molecules and the atomic Debye–Waller parameters. In principle, all CTRs are sensitive to the presence of organothiol molecules in the model, the CTRs with low momentum transfer disclose the out-of-plane electron density, and the remaining CTRs can reveal in-plane information.

Calcite growth

Calcium carbonate crystals were grown at room temperature on top of both potassium and copper terminated mica covered with organothiols using the 'ammonium carbonate' method, as was described by Aizenberg et al. [9] In short, K-mica and Cu-mica surfaces containing organothiols were placed upside down in a 24 well plate (ThermoFisher Scientific) submerged in a 10^{-2} M $CaCl_2$ solution. This setup was then placed in a closed desiccator containing 1.5 g of ammonium carbonate. After approximately 48 hours, the samples were washed three times in demineralized water for at least 30 seconds and dried with a gentle flow of nitrogen. The crystallization was repeated on a different day in a fresh batch, to demonstrate reproducibility of the experiment.

A different method to grow calcium carbonate crystals was described by Park and Meldrum [1, 10]. In this 'sodium carbonate' method, an aqueous solution of 10^{-2} M calcium chloride dihydrate was added to an aqueous solution of 10^{-2} M sodium carbonate in equal amounts at room temperature. Both K-mica and Cu-mica with thiols were placed upside down in a 24 well plate (ThermoFisher Scientific) submerged in this solution for 2 hours. Subsequently, the samples were washed three times in demineralized water for at least 30 seconds and dried with a gentle flow of nitrogen.

In order to weather muscovite mica, it was exposed to 43% relative humidity for 1.5 h by placing the samples in a closed compartment containing a separate vial of a saturated solution of potassium carbonate held at room temperature.

Scanning electron microscopy images were acquired using a PhenomWorld Phenom Scanning Electron Microscope. For these measurements, a thin gold layer was applied to the samples using a Cressington 108autosputter coater.

Ion-exchange procedure, XPS setup specifications, and XPS spectrum of Cu-terminated muscovite mica

Muscovite mica was freshly cleaved and placed into an aqueous solution of 10^{-3} M of Cu(II)chloride, dihydrate (99% pure, obtained from Merck) for at least one hour for ion exchange. This solution was filtered prior to use with a 0.2 μm pore size Whatman filter, to remove any large crystallites and other

particles from the solution. The sample was subsequently washed three times in approximately 15 mL of water (ultrapure, 18.2 MΩ/cm resistance and < 3 ppb organic content (MQ)) for approximately 1 minute. The sample was then directly used for the organothiol functionalization.

X-ray photoelectron spectroscopy (XPS) was performed at the ESRF, using an aluminium anode as an X-ray source of 1486.6 eV, a hemispherical electron energy analyser with channeltron, and a base pressure of the ultra-high vacuum chamber of $5 \cdot 10^{-10}$ mbar. The spectrum of Cu-terminated muscovite mica is shown in Figure S20. Clear peaks are visible coming from the copper $2p_{1/2}$ and $2p_{3/2}$ photoelectron lines. The intensity of these peaks was integrated with a linear baseline correction and normalized to the K 2s peak (see Table S3). The higher normalized intensity at an angle of 15° with respect to 45° shows that the copper ions are located at the surface of muscovite mica.

Table S3 Normalized peak intensities of the Cu $2p_{3/2}$ peak with respect to the K 2s peak

Peak measured at 15° exit angle	Normalized intensity (counts)	Peak measured at 45° exit angle	Normalized intensity (counts)
Cu $2p_{3/2}$	$7 \cdot 10^{3}$	Cu $2p_{3/2}$	1.7

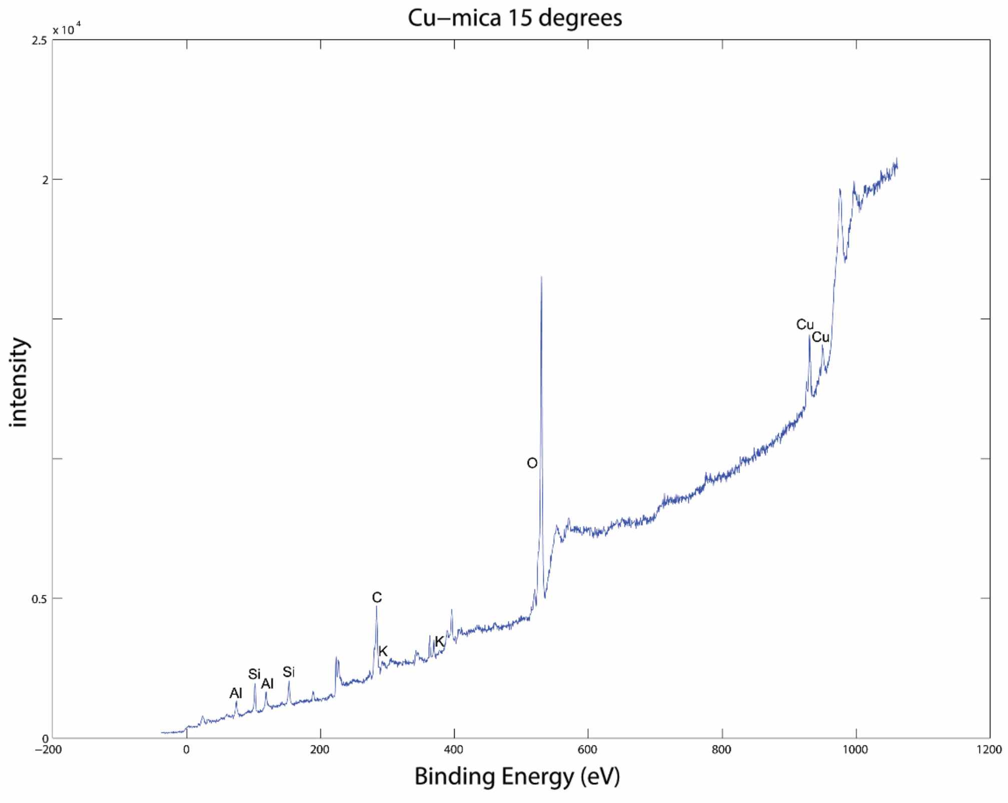

Figure S24 XPS spectrum of Cu-terminated muscovite mica, measured at 15° exit angle.

References

[1] C.J. Stephens, Y. Mouhamad, F.C. Meldrum, H.K. Christenson, Epitaxy of Calcite on Mica, Cryst. Growth Des., 10 (2010) 734-738.

[2] C.D. Bain, Sum-frequency vibrational spectroscopy of the solid-liquid inteface, J. Chem. Soc.-Faraday Trans., 91 (1995) 1281-1296.

[3] A.N. Parikh, D.L. Allara, Quantitative determination of molecular structure in multilayered thin-films of biaxial and lower symmetry from photon spectroscopies. 1. Reflection infrared vibrational spectroscopy., J Chem Phys, 96 (1992) 927-945.

[4] K.A. Link, G.N. Spurzem, A. Tuladhar, Z. Chase, Z.M. Wang, H.F. Wang, R.A. Walker, Organic Enrichment at Aqueous Interfaces: Cooperative Adsorption of Glucuronic Acid to DPPC Monolayers Studied with Vibrational Sum Frequency Generation, J. Phys. Chem. A, 123 (2019) 5621-5632.

[5] W. de Poel, S.L. Vaessen, J. Drnec, A.H.J. Engwerda, E.R. Townsend, S. Pintea, A.E.F. de Jong, M. Jankowski, F. Carla, R. Felici, J. Elemans, W.J.P. van Enckevort, A.E. Rowan, E. Vlieg, Metal ion-exchange on the muscovite mica surface, Surf. Sci., 665 (2017) 56-61.

[6] E. Vlieg, Integrated intensities using a six-circle surface X-ray diffractometer, J. Appl. Crystallogr., 30 (1997) 532-543.

[7] E. Vlieg, ROD, a program for surface crystallography, J. Appl. Crystallogr., 33 (2000) 401-405.

[8] W. de Poel, S. Pintea, J. Drnec, F. Carla, R. Felici, P. Mulder, J. Elemans, W.J.P. van Enckevort, A.E. Rowan, E. Vlieg, Muscovite mica: Flatter than a pancake, Surf. Sci., 619 (2014) 19-24.

[9] J. Aizenberg, A.J. Black, G.M. Whitesides, Oriented Growth of Calcite Controlled by Self-Assembled Monolayers of Functionalized Alkanethiols Supported on Gold and Silver, J. Am. Chem. Soc., 121 (1999) 4500-4509.

[10] R.J. Park, F.C. Meldrum, Shape-constraint as a route to calcite single crystals with complex morphologies, J. Mater. Chem., 14 (2004) 2291-2296.
